# Psychiatric comorbidities with autism spectrum disorder in an adult clinic sample

**DOI:** 10.1192/bjo.2021.639

**Published:** 2021-06-18

**Authors:** Rifat Binte Radwan, Chiro Islam Mallik

**Affiliations:** 1 Royal Victoria Infirmary, Cumbria, Northumberland, Tyne and Wear NHS Foundation trust; 2 Community Learning Disability Team, Hertfordshire Partnership University NHS Foundation Trust

## Abstract

**Aims:**

As part of continuity, prevalence of Autism Spectrum Disorder (ASD) is nearly the same in adults as children and is associated with other comorbid psychiatric disorders that have substantial impact on their life and complex the intervention. This study aimed to examine psychiatric comorbidity in referred adult ASD patients compared to non-ASD psychiatric patients. It has been hypothesized that comorbid psychiatric disorders were higher among patients with ASD than patients without ASD.

**Method:**

In total, 36 adults with ASD referred in the year 2019 in a psychiatric consultation center in Dhaka city were included in the study. They were derived from the case register of the center. Similar number of age and sex-matched adult psychiatric patients without ASD were selected for comparison. All patients were referred for psychiatric consultation. Socio-demographic variables were collected from the patients’ record. Diagnosis of psychiatric disorders including ASD was made by an experienced psychiatrist. It was done clinically based on all available information, examination and relevant investigations. Diagnoses were assigned according to DSM-5. Then comparisons of psychiatric disorders were made between the two patient groups.

**Result:**

The cases were ranged from 18-41 years with the mean of 26.72 ± 6.5 years. Among them, 22 were male and 14 were female. Male-female ratio was 1.6:1. Most of the subjects received no education and were from middle income family with urban background. Mean number of comorbid psychiatric disorders was 1.92 in patients with ASD and 1.67 in patients without ASD and the difference was significant (P = 0.04). Most two frequent comorbidities among ASD patients were Obsessive Compulsive Disorder (27.77%) and Major Depressive Disorder(25%) followed by Specific Phobia(19.44%), Social Phobia and Intermittent Explosive Disorder(16.67%) for each, Attention Deficit Hyperactivity Disorder(13.89%) and Conduct Disorder(11.11%). All these disorders were significantly higher than patients without ASD. Conversely, Major Depressive Disorder (30.55%) was most frequent among the patients without ASD and that was even significantly higher than patients with ASD. Other frequent disorders like Bipolar Disorder, Schizophrenia, Generalized Anxiety Disorder and Substance Related Disorder were also higher among non-ASD patients.

**Conclusion:**

This research shows that comorbid psychiatric disorders were frequently found in patients with ASD. Subsequent broad-based studies using extensive measures of psychopathology are required to confirm these preliminary findings. Greater understanding of the presence of other psychiatric disorders in ASD patients will turn this awareness into action.

